# Cohort Differences in Depressive Symptoms and Life Satisfaction in 75- and 80-Year-Olds: A Comparison of Two Cohorts 28 Years Apart

**DOI:** 10.1177/08982643231164739

**Published:** 2023-03-22

**Authors:** Tiia Kekäläinen, Kaisa Koivunen, Katja Pynnönen, Erja Portegijs, Taina Rantanen

**Affiliations:** 1 Gerontology Research Center and Faculty of Sport and Health Sciences, 4168 University of Jyväskylä, Jyväskylä, Finland; 2 Human Movement Sciences, University Medical Center Groningen, 505889 University of Groningen, The Netherlands

**Keywords:** mental health, depression, secular trends, birth cohorts

## Abstract

**Objectives:** To examine birth cohort differences in depressive symptoms and life satisfaction in older men and women and the mechanisms underpinning the possible cohort differences. **Methods:** Two independent cohorts of Finnish men and women aged 75 and 80 were assessed in 1989–1990 (*n* = 617) and 2017–2018 (*n* = 794). They reported their depressive symptoms (CES-D), current life satisfaction, and evaluation of life until now. **Results:** The later-born cohort reported fewer depressive symptoms (8.6 ± 7.1 vs. 13.9 ± 8.3) and the differences were similar for the subdomains of depressive symptoms. The later-born cohort was more often mostly satisfied with life until now (90 vs. 70%) but not with the current life than the earlier-born cohort. Better self-rated health and education of the later-born cohort partly explain the cohort differences. **Discussion:** Older people in Finland report fewer depressive symptoms and they are more satisfied with their past life compared to their counterparts assessed 28 years ago.

## Introduction

Life expectancy has increased in the last decades and older people today have better physical and cognitive functioning than older people a couple of decades ago (e.g., Beller et al., 2022; Christensen et al., 2013; Koivunen et al., 2021; Munukka et al., 2021; Strand et al., 2019; Öhman et al., 2022). In Finland, the increase in life expectancy has been exceptionally large during the 20^th^ century compared to other OECD countries (Kangas, 2010). Finland was hit severely first in the civil war (1918) and later in World War II. The life expectancy in Finland was among the lowest in OECD countries at the beginning of the 20^th^ century and during World War II but reached the other Nordic countries by the end of the 20^th^ century (Kangas, 2010). After World War II, societal development and reforms have improved material conditions and overall living standards in most countries. The social reforms in Finland included, for example, the provision of free school meals for all children, better access to education, and the overall industrialization of society. These changes are reflected in many areas of life, such as development in technology and medical science, increase in average educational level, improved working conditions, and changes in life habits, that shape adult development and aging (Drewelies et al., 2019).

Many of these changes have occurred in the areas of life that are related to mental well-being. For example, low education, poverty, substance use, physical inactivity, presence of chronic diseases, cognitive impairments, and poor physical functioning are risk factors for depression (Zenebe et al., 2021) and poor life satisfaction (Burton-Jeangros & Zimmermann-Sloutskis, 2016; Capone et al., 2021; Pynnönen et al., 2021) in old age. Thus, the changes in society during the last decades may affect not only the different domains of health and functioning but also the experience of life.

Mental well-being is an important outcome as such, but it also contributes to many crucial areas of life. Good mental well-being predicts longevity, better health, and better overall functioning (Diener & Chan, 2011; Formánek et al., 2020; Kokko & Feldt, 2018; Walker, 2005). Mental well-being includes cognitive and affective evaluations of life (Diener, 1984). The affective side of mental well-being represents the existence of both pleasant and unpleasant affect while the cognitive evaluations of life as a whole are captured by life satisfaction (Diener, 1984; Diener et al., 1999).

While the overall improvement in living conditions and health and functioning may be reflected in more positive experiences of life, evaluations of life are not based only on life circumstances but also on adaptation and comparison. Even though the likelihood of some physical and social losses, such as functional limitations and widowhood, increases with age, older adults seem to maintain and even increase their level of mental well-being (Diener et al., 1999; Sutin et al., 2013). According to the so-called “satisfaction paradox,” individuals accommodate themselves to their current circumstances and lower their expectations if needed (Walker, 2005). Thus, older cohorts may be more satisfied than younger cohorts would be in the same circumstances. Exceeding the earlier expectations concerning own life leads to better life satisfaction (Diener & Fujita, 1997). The cohorts that have lived through rougher times, such as wars, may have adjusted their point of reference low leading to good satisfaction with the current situation (de Ree & Alessie, 2011). The improvements in circumstances over a period of time may elevate standards for higher life satisfaction, leading to life satisfaction of subsequent birth cohorts remaining at the same level (Henning et al., 2022).

Research results on cohort differences in mental well-being have been inconsistent due to, for example, differences in the assessed dimension of mental well-being, participants’ age, and the time between cohorts. Positive secular changes have been found for positive affect among U.S. adults (Sutin et al., 2013), German older adults (Gerstorf et al., 2015), and German adults in retirement transition (Henning et al., 2022), and general mental well-being in Finnish older adults (Öhman et al., 2022). However, no cohort differences have been found for life satisfaction (Henning et al., 2022; Jivraj et al., 2014) and depressed affect (Sutin et al., 2013). The secular change in depression seems to be even the opposite as the prevalence of both diagnosed depression and depressive symptoms has increased among middle-aged adults in various countries (Abrams & Mehta, 2019; Bishop et al., 2022; Jeuring et al., 2018; Spiers et al., 2012). The evidence for older adults (70+) is less consistent: the studies suggest either no cohort differences in the prevalence of depressive symptoms (Abrams & Mehta, 2019; Timmermans et al., 2019) and major depression (Wiberg et al., 2013) but in some studies, there has been an increase in the prevalence of depressive symptoms among 70–79 years old adults (Bishop et al., 2022) and minor depression among 75 years old adults (Wiberg et al., 2013).

Altogether, these results suggest that while later-born cohorts perceive more positive feelings than earlier-born cohorts, the same positive change may not be seen in overall life satisfaction and depressive symptoms. In addition, the mechanisms that underpin cohort differences in mental well-being are hardly studied. We investigated differences in depressive symptoms and life satisfaction in independent cohorts of 75 and 80 years old Finnish community-dwelling older adults born 28 years apart. In addition, we investigated whether improvements in socioeconomic and social resources, as well as better functional capacity, explain the potential cohort differences in mental well-being.

## Methods

### Recruitment and Participation

The Evergreen I and Evergreen II recruitment procedures are comparable, and recruitment was as inclusive as possible. The recruitment processes are described in more detail in study protocols (Heikkinen, 1998; Rantanen et al., 2018) and in previous cohort comparisons (Koivunen et al., 2021; Munukka et al., 2021). Samples were drawn from the Finnish population registers based on birth year and place of residence. The target group was all 75- and 80-year-old persons who were living in the recruitment area in a non-institutional setting. Members of the earlier cohort were born in 1910 and 1914, and in the later cohort in 1938–1939 and 1942–1943.

In the Evergreen I study, conducted in 1989–1990, an information letter of the study and a suggested time for a home interview were sent to all potential participants in the target group. Those who declined were asked to report their reasons for non-participation. In the Evergreen II, in 2017–2018, all potential participants were invited to participate in the study 2017, and an additional randomly selected half of the target group was invited in 2018. Participants were first sent an information letter about the study and then their willingness to participate was asked by phone. For those willing to participate, a home interview was scheduled during the phone call, and for those who declined to participate, the reason for non-participation was asked. In the Evergreen I study, of the eligible participants (*n* = 652), 95% (*n* = 617) participated in at least the home interview and 77% (*n* = 500) in both the home interview and laboratory measurements. In the Evergreen II study, the corresponding proportions were 43% (*n* = 794) and 40% (*n* = 726) of the eligible participants (*n* = 1835) (Portegijs et al., 2019). All who participated in the home interview were included in the present study.

All participants in the Evergreen I and II studies signed an informed consent, and the research ethical principles required at the time were followed. The Evergreen II study was approved by the ethical committee of the Central Finland Hospital (August 23, 2017).

Our earlier analyses indicated that non-participants in earlier and later cohorts were comparable (Koivunen et al., 2021). Poor self-rated health was somewhat less common in the non-participants of the later-born cohort than the earlier-born cohort (17 vs. 23 %) and lack of interest or time was more common (48 vs. 40%, correspondingly).

### Variables

*Depressive symptoms* were assessed by the Center for the Epidemiological Studies Depression Scale (CES-D) (Radloff, 1977). Participants are asked to report their feelings and experiences during the past week with 20 items. The scale contains four dimensions: depressed affect (7 items, e.g., “I felt sad” and “I thought my life had been a failure”), positive affect (4 items, e.g., “I enjoyed life” and “I felt hopeful about the future”), somatic symptoms (7 items, e.g., “My sleep was restless” and “I could not get going”), and interpersonal problems (2 items, “People were unfriendly” and “I felt that people dislike me”). The response scale was Seldom or never (=0), Sometimes (=1), Rather often (=2), and Almost all the time (=3). After reverse coding of the reverse items, the sum scores for 20 items and four dimensions were calculated for participants with at most one missing item. A higher value represents more depressive symptoms. Cronbach´s alphas were .85 at age 75, .84 at age 80 for the Evergreen I cohort and .87 at age 75, and .89 at age 80 years for the Evergreen II cohort.

*Life satisfaction* was studied using two questions. The current situation was rated with the question “Are you happy and satisfied with your life?” with response options No (=1); Yes, occasionally (=2); and Yes, usually (=3). The satisfaction during the life course was asked with the question “How would you evaluate your life until now?” with response options Mostly unsatisfactory (=1), Occasionally unsatisfactory (=2), and Mostly satisfactory (=3). Due to a low number of replies (<2%) in the lowest categories, the two most negative categories were combined for the analysis of both items.

### Covariates

The analyses were not adjusted for any confounders. The age and sex groups were similar, and differences between cohorts in other variables were more likely to be factors contributing to the cohort differences than confounders. To study these factors, correlates of depressive symptoms and life satisfaction that differed between the cohorts were chosen for the analyses.

Social resources were assessed by marital status (married vs. not married), social contacts, and loneliness. Social contacts were assessed with three questions asking the frequency to meet (a) children or other relatives, (b) close friends, and (c) other acquaintances with a response scale from I do not have any (=0) to every day (=5). The sum score from the three items was calculated. Loneliness was assessed with a single item: “How often do you feel lonely?” with a response scale of very rarely/never (=1), rarely (=2), often (=3), and almost always (=4) (Tiikkainen & Heikkinen, 2005). The category “almost always” was merged with the category “often” due to a low number of replies (1%) in the highest category. Socioeconomic position was expressed by full years of education. Self-rated health was asked with a response scale from very good (=1) to very poor (=5) (Heikkinen et al., 2011).

Functional capacity measures were available for 81% of Evergreen I and 91% for Evergreen II samples, who took part also in physical and cognitive tests. Maximum walking speed was assessed using a ten-meter test in the laboratory corridor. Participants were instructed to walk as fast as possible, and the time was taken with a hand-held stopwatch. Cognitive functioning was assessed using the Digit Symbol coding task from Wechsler Adult Intelligence Scale-Revised (Wechsler & De Lemos, 1981). In the test, participants draw the correct symbols below their equivalent numbers by using a number-to-symbol coding key. The test measures processing speed, working memory, visuospatial processing, and attention.

### Statistical Analyses

To compare the earlier and current same-age cohorts, t-tests for continuous and chi-square tests for categorical variables were used.

The factors underlying the cohort differences were tested with a set of regression models. Linear regression for CES-D and its four dimensions and binary logistic regression for two life satisfaction items were used. The first model included only the birth cohort as an independent variable. Then several models were run adding covariates one at a time to see which of them attenuates the cohort differences. Finally, models including all five covariates simultaneously were estimated. For linear regression models, the change (%) in regression coefficients between models was calculated to present how much covariate(s) explained from the potential cohort difference. A similar procedure was used in additional analyses for the smaller laboratory sample to see whether walking speed and cognitive functioning attenuate the cohort differences.

Coefficients from nested binary logistic regression models are not directly comparable because of a rescaling of the models (Karlson et al., 2012). The Karlson‒Holm‒Breen (KHB) method in R version 2022.12.0 using package *khb* version .11 (Karlson et al., 2012) was used to provide comparable coefficients for a model including only the birth cohort and a model including the birth cohort and covariate(s). Additionally, the KHB quantifies the extent that controlling for covariates attenuated the association between the cohort and life satisfaction and provides a test for the statistical significance of the difference in the coefficients across models.

## Results

Descriptive statistics are shown in Table 1. The later-born cohort had more years of education, better walking speed, and better self-rated health in both age groups and both men and women, as reported before with a sample comprising solely those who took part in the laboratory tests (Koivunen et al., 2021; Munukka et al., 2021). Among women and 80-year-old men, a larger proportion of the later-born cohort was married than of the earlier-born cohort. Among 75-year-old men and 80-year-old women, the later-born cohort reported more frequent social contacts and among 75-year-old men and women, the later-born cohort reported being lonely less often than the earlier cohort. The opposite was true for 80-year-old women.

**Table 1.** Descriptive Statistics and Cohort Differences of 75- and 80-Year-Old Men and Women Born in 1910 and 1914 (Evergreen Cohort) and Born in 1938–1939 and 1942–1943 (Evergreen II Cohort).

	75 years			80 years		
	1989–1990	2017–2018	Cohort difference	1989–1990	2017–2018	Cohort difference
	M (SD)	M (SD)	*p*-value	M (SD)	M (SD)	*p*-value
**Men**						
*N*	119	193		74	149	
Years of education	6.3 (3.5)	12.2 (4.3)	<.001	6.0 (4.3)	11.8 (4.4)	<.001
Walking speed	1.8 (.5)	2.0 (.4)	<.001	1.5 (.5)	1.8 (.4)	<.001
Social contacts	9.4 (3.0)	10.1 (1.9)	.025	9.0 (3.3)	9.9 (2.2)	.053
Self-rated health, *n* (%)			<.001			.002
Very good/good	15 (13.6)	109 (56.5)		13 (19.1)	64 (43.2)	
Average	83 (75.5)	77 (39.9)		39 (57.4)	80 (54.1)	
Poor/very poor	12 (10.9)	7 (3.6)		16 (23.5)	4 (2.7)	
Marital status, *n* (%)			.783			.005
Married	99 (83.2)	162 (84.4)		51 (68.9)	126 (85.1)	
Not married	20 (16.8)	30 (15.6)		23 (31.1)	22 (14.9)	
Loneliness, *n* (%)			.004			.053
Very rarely or never	69 (58.5)	134 (70.5)		54 (78.3)	90 (61.6)	
Rarely	40 (33.9)	54 (28.4)		12 (17.4)	46 (31.5)	
Often or almost always	9 (7.6)	2 (1.1)		3 (4.3)	10 (6.8)	

**Women**						
*N*	236	265		188	187	
Years of education	6.0 (3.2)	12.0 (4.1)	<.001	5.6 (3.2)	11.6 (5.9)	<.001
Walking speed	1.5 (.4)	1.7 (.3)	<.001	1.2 (.3)	1.6 (.3)	<.001
Social contacts	10.0 (2.6)	10.0 (2.1)	.962	9.0 (2.8)	10.0 (2.0)	<.001
Self-rated health, *n* (%)			<.001			<.001
Very good/good	32 (15.4)	142 (53.8)		27 (15.0)	79 (42.5)	
Average	149 (71.6)	113 (42.8)		113 (62.8)	98 (52.7)	
Poor/very poor	27 (13.0)	9 (3.4)		40 (22.2)	9 (4.8)	
Marital status, *n* (%)			<.001			<.001
Married	51 (21.6)	139 (52.7)		34 (18.1)	83 (44.6)	
Not married	185 (78.4)	125 (47.3)		154 (81.9)	103 (55.4)	
Loneliness, *n* (%)			.008			<.001
Very rarely or never	135 (59.7)	141 (53.8)		127 (70.6)	94 (50.5)	
Rarely	63 (27.9)	104 (39.7)		35 (19.4)	76 (40.9)	
Often or almost always	28 (12.4)	18 (6.8)		18 (10.0)	16 (8.6)	

*Note.* Cohort differences were tested with an independent samples t-test for years of education, walking speed, and social contacts, and with a chi-squared test for self-rated health, marital status, and loneliness.

The differences between the cohorts in depressive symptoms and life satisfaction are shown in Table 2. Consistently across genders and age groups, the later-born cohort reported fewer depressive symptoms than the earlier-born cohort (the differences 4.9–5.7 points). The differences between cohorts were seen in all subscales of CES-D except for nonsignificant cohort differences in interpersonal problems among 75-year-old men and somatic symptoms among 80-year-old men. The later-born cohort perceived their life satisfaction until now better than the earlier-born cohort. There were no cohort differences in the current life satisfaction, except that among 80-year-old men the earlier-born cohort perceived their current life satisfaction better than the later-born cohort.

**Table 2.** Cohort Differences in Depressive Symptoms and Life Satisfaction of 75- and 80-Year-Old Men and Women Born in 1910 and 1914 (Evergreen Cohort) and Born in 1938–1939 and 1942–1943 (Evergreen II Cohort), Respectively.

	75 years			80 years		
	1989–1990	2017–2018	*p*-value	1989–1990	2017–2018	*p*-value
	M (SD)	M (SD)		M (SD)	M (SD)	
**Men**						
Depressive symptoms	11.9 (7.2)	7.0 (6.1)	<.001	13.5 (8.7)	8.4 (7.5)	<.001
Depressed affect	2.6 (2.9)	1.2 (2.0)	<.001	3.2 (3.5)	1.6 (2.5)	.002
Lack of positive affect	5.0 (2.9)	2.7 (2.4)	<.001	4.5 (3.4)	2.9 (2.5)	<.001
Somatic symptoms	3.9 (3.0)	2.8 (2.6)	.001	4.4 (3.4)	3.5 (3.3)	.073
Interpersonal problems	.4 (.8)	.3 (.7)	.495	.9 (1.3)	.4 (.7)	.004
Current life satisfaction, f (%)			.594			.014
Lower	12 (11.2)	16 (8.4)		3 (4.4)	24 (16.3)	
Higher	106 (89.8)	175 (91.6)		65 (95.6)	123 (83.7)	
Life satisfaction until now, f (%)			<.001			<.001
Lower	34 (28.8)	14 (7.4)		22 (33.3)	14 (9.5)	
Higher	84 (71.2)	176 (92.6)		44 (66.7)	133 (90.5)	

**Women**						
Depressive symptoms	14.3 (8.6)	8.6 (7.0)	<.001	14.8 (8.3)	9.1 (7.3)	<.001
Depressed affect	3.8 (3.9)	1.2 (2.7)	<.001	3.8 (3.4)	2.2 (2.8)	<.001
Lack of positive affects	5.6 (3.0)	2.9 (2.4)	<.001	5.7 (3.4)	3.2 (2.7)	<.001
Somatic symptoms	4.5 (3.7)	3.5 (2.7)	.001	4.7 (3.5)	3.5 (2.9)	.001
Interpersonal problems	.5 (1.0)	.3 (.8)	.021	.6 (1.0)	.2 (.5)	<.001
Current life satisfaction, f (%)			.145			.357
Lower	26 (11.5)	20 (7.6)		26 (14.4)	21 (11.2)	
Higher	201 (88.5)	243 (92.4)		154 (85.6)	166 (88.8)	
Life satisfaction until now, f (%)			<.001			<.001
Lower	61 (26.9)	26 (9.9)		59 (32.8)	18 (9.8)	
Higher	166 (73.1)	237 (90.1)		121 (67.2)	166 (90.2)	

*Note.* Cohort differences were tested with an independent samples t-test for depressive symptoms total score and dimensions, and with a chi-squared test for life satisfaction. Depressive symptoms possible range 0–60 and for dimensions: depressed affect 0–21, positive affects 0–12, somatic symptoms 0–21, and interpersonal problems 0–6.

The results of regression analyses are shown in Table 3 for depressive symptoms, Tables 4 and 5 for life satisfaction, and CES-D subscales in supplementary materials (Supplementary Tables S1 and S2). The strongest explanatory variable of cohort differences was the higher self-rated health in the later-born cohort, which explained 36–49% of the cohort differences in depressive symptoms. Among men, the higher education in the later-born cohorts explained 19–23% of the difference. Controlling simultaneously for education, marital status, social contacts, loneliness, and self-rated health attenuated the associations between birth cohort and depressive symptoms for 30% in women, 54% in 75-year-old men, and 43% in 80-year-old men, but the associations remained statistically significant. The results for CES-D subscales were similar to those for the total CES-D scale: the more favorable values of the later-born cohorts in the CES-D subscales were explained by their more favorable values in self-rated health and in men in education. The association between birth cohorts and CES-D subscales of depressed affect, lack of positive affect, and interpersonal problems remained statistically significant after all covariates were added in the models. The cohort differences in the somatic symptoms subscale were attenuated after adjusting the models for self-rated health.

**Table 3.** Linear Regression Coefficients of the Association Between Birth Cohort and Depressive Symptoms.

	75 years				80 years			
	Birth cohort			Model	Birth cohort			Model
	B	SE	*p*	Adj R2	B	SE	*p*	Adj R2
Men								
Birth cohort	−5.57	.89	<.001	.11	−6.24	1.49	<.001	.08
+Education	−4.49	1.09	<.001	.12	−4.80	1.76	.007	.08
+Marriage	−5.52	.87	<.001	.15	−5.48	1.49	<.001	.10
+Social contacts	−5.44	.90	<.001	.11	−5.75	1.59	<.001	.09
+Loneliness	−4.59	.85	<.001	.22	−7.76	1.34	<.001	.28
+Self-rated health	−3.58	.95	<.001	.18	−3.21	1.47	.030	.22
+All	−2.55	1.09	.020	.28	−3.57	1.80	.048	.35

Women								
Birth cohort	−6.08	.76	<.001	.12	−6.40	.96	<.001	.12
+Education	−5.56	.98	<.001	.12	−7.20	1.14	<.001	.12
+Marriage	−6.04	.80	<.001	.11	−5.90	1.00	<.001	.12
+Social contacts	−6.09	.75	<.001	.13	−5.82	1.01	<.001	.14
+Loneliness	−6.06	.70	<.001	.25	−7.08	.92	<.001	.20
+Self-rated health	−3.37	.80	<.001	.22	−3.94	.95	<.001	.23
+All	−4.24	.99	<.001	.30	−4.47	1.32	<.001	.28

*Note.* B = Unstandardized beta indicates mean cohort difference (reference group Evergreen cohort), SE = standard error, Adj R^2^ = adjusted R^2^ (linear regression). Each covariate was added in the model one at a time with birth cohort and all five together in the model “All.”

**Table 4.** Logistic Regression Coefficients of the Association Between Birth Cohort and Life Satisfaction in Men.

	Men 75 years				Men 80 years			
	Birth cohort			Model	Birth cohort			Model
	B	SE	*p*	R2	B	SE	*p*	R2
Current life satisfaction								
Birth cohort	.23	.40	.564	.00	−1.35	.64	.034	.05
+Education	.33	.49	.498	.00	−1.91	.71	.007	.08
Birth cohort	.21	.40	.604	.00	−1.61	.65	.013	.06
+Marriage	.21	.40	.607	.01	−1.87*	.67	.005	.15
Birth cohort	.18	.41	.661	.00	−1.58	.65	.015	.07
+Social contacts	.09	.42	.837	.02	−1.72	.67	.010	.10
Birth cohort	.13	.41	.760	.00	−1.65	.69	.017	.06
+Loneliness	−0.08*	.43	.845	.08	−1.28*	.68	.061	.31
Birth cohort	.14	.43	.740	.00	−1.78	.69	.010	.06
+Self-rated health	−0.35*	.45	.436	.05	−2.64**	.78	.001	.22
Birth cohort	.05	.45	.918	.00	−1.56	.73	.032	.06
+All	−0.35	.56	.524	.14	−2.22	.90	.013	.38

Life satisfaction until now								
Birth cohort	1.65	.35	<.001	.13	1.64	.39	<.001	.14
+Education	1.10	.43	.010	.15	1.19	.49	.015	.15
Birth cohort	1.62	.34	<.001	.13	1.56	.39	<.001	.13
+Marriage	1.62	.34	<.001	.13	1.46	.39	<.001	.14
Birth cohort	1.63	.34	<.001	.13	1.64	.41	<.001	.14
+Social contacts	1.61	.35	<.001	.13	1.55	.42	<.001	.17
Birth cohort	1.63	.35	<.001	.13	1.66	.40	<.001	.13
+Loneliness	1.54	.35	<.001	.15	1.83	.42	<.001	.19
Birth cohort	1.61	.36	<.001	.12	1.63	.40	<.001	.13
+Self-rated health	1.22*	.37	.001	.15	1.09**	.42	.008	.21
Birth cohort	1.65	.36	<.001	.12	1.94	.46	<.001	.15
+All	.70**	.46	.123	.18	1.19	.60	.046	.26

*Note.* B = KHB logistic regression coefficients indicating cohort difference (reference group Evergreen cohort), SE = standard error, R^2^ = Pseudo R^2^. *p < .05, **p < .01, ***p < .001 indicating a statistically significant difference in KHB logistic regression coefficients between a model with only a birth cohort and a model with covariate(s). Each covariate was added in the model one at a time with birth cohort and all five together in the model “All.”

**Table 5.** Logistic Regression Coefficients of the Association Between Birth Cohort and Life Satisfaction in Women.

	Women 75 years				Women 80 years			
	Birth cohort			Model	Birth cohort			Model
	B	SE	*p*	R2	B	SE	*p*	R2
Current life satisfaction								
Birth cohort	.57	.33	.088	.01	.26	.32	.417	.00
+Education	−0.01	.44	.982	.03	.37	.37	.322	.01
Birth cohort	.50	.32	.112	.01	.31	.32	.335	.00
+Marriage	.51	.33	.124	.01	.12	.33	.723	.02
Birth cohort	.47	.31	.143	.01	.11	.32	.724	.00
+Social contacts	.48	.32	.139	.06	.03	.33	.938	.01
Birth cohort	.45	.34	.177	.01	.29	.33	.380	.00
+Loneliness	.46	.34	.172	.20	.47*	.33	.149	.11
Birth cohort	.52	.33	.115	.00	.31	.33	.341	.00
+Self-rated health	−0.02***	.35	.965	.08	−0.40***	.36	.264	.16
Birth cohort	.46	.37	.217	.01	.20	.36	.588	.00
+All	−0.61*	.53	.252	.25	−0.39	.46	.402	.19

Life satisfaction until now								
Birth cohort	1.30	.27	<.001	.08	1.46	.29	<.001	.12
+Education	.68*	.34	.044	.11	1.34	.36	<.001	.12
Birth cohort	1.21	.26	<.001	.08	1.50	.30	<.001	.12
+Marriage	1.18	.27	<.001	.08	1.42	.30	<.001	.13
Birth cohort	1.21	.26	<.001	.08	1.42	.31	<.001	.11
+Social contacts	1.22	.26	<.001	.12	1.32	.31	<.001	.13
Birth cohort	1.25	.26	<.001	.08	1.60	.31	<.001	.12
+Loneliness	1.25	.26	<.001	.15	1.76*	.31	<.001	.20
Birth cohort	1.27	.26	<.001	.08	1.56	.30	<.001	.13
+Self-rated health	.87**	.28	.002	.12	1.26**	.31	<.001	.16
Birth cohort	1.39	.28	<.001	.09	1.49	.32	<.001	.11
+All	.46**	.38	.218	.20	1.32	.45	.003	.20

*Note.* B = KHB logistic regression coefficients indicating cohort difference (reference group Evergreen cohort), SE = standard error, R^2^ = Pseudo R^2^. *p < .05, **p < .01, ***p < .001 indicating a statistically significant difference in KHB logistic regression coefficients between a model with only a birth cohort and a model with covariate(s). Each covariate was added in the model one at a time with birth cohort and all five together in the model “All.”

The birth cohort difference in current life satisfaction among 80-year-old men favored the earlier-born cohort which coincided with their slightly less frequent reports of loneliness (Table 4). The advantage in the current life satisfaction in the earlier-born 80-year-old men became even higher after adjusting for their lower proportion being married and higher proportion reporting poorer self-rated health.

The associations between birth cohort and life satisfaction until now were statistically significantly attenuated after adjusting the models for self-rated health in both men and women and both age groups, and among 75-year-old women also after adjusting the model for education (Tables 4 and 5): the more favorable evaluations of the past life in the later-born cohorts were explained by their more favorable values in self-rated health and education. The birth cohort differences in life satisfaction until now remained statistically significant even after controlling for education, marital status, social contacts, loneliness, and self-rated health simultaneously among 80-year-olds but not among 75-year-olds.

The additional analyses conducted among those who took part also in the laboratory tests are shown in supplementary materials (Supplementary Tables S3–S6). The cohort differences in mental well-being favoring the later-born cohort remained statistically significant after adjusting the models for cognitive functioning and walking speed.

## Discussion

The present study investigated cohort differences in mental well-being in 75- and 80-year-old men and women born 28 years apart. The results showed that the later-born cohorts reported fewer depressive symptoms and better life satisfaction during the life course compared to the earlier-born cohorts consistently among men and women and 75- and 80-year-old people. The cohort differences remained significant even after controlling for education, social resources, self-rated health, and functional performance. The same positive cohort difference was not seen in the current life satisfaction.

Our findings are in line with those previous studies that have suggested that older adults today have better mental well-being than people of the same age assessed two to three decades ago (Henning et al., 2022; Gerstorf et al., 2015; Sutin et al., 2013; Öhman et al., 2022). In addition, the current results further support the idea of improvements especially on the affective side of mental well-being (Gerstorf et al., 2015; Henning et al., 2022; Sutin et al., 2013). In the present study, later-born cohorts reported fewer depressive symptoms. The difference between cohorts was around five points, which is also the threshold for the minimum clinically important difference in depressive symptoms (Masson & Tejani, 2013). Of the CES-D subscales, the most consistent cohort differences were seen in positive affect and depressive affect. The functioning and development of individuals, including their experiences of emotions and emotion regulation, are shaped by the sociocultural environment they grow up in and the resources available to them throughout their lives (Drewelies et al., 2019).

At the same time, our results do not lend support to the previous findings showing an increase in the prevalence of depression and depressive symptoms across countries (Abrams & Mehta, 2019; Bishop et al., 2022; Jeuring et al., 2018; Spiers et al., 2012; Wiberg et al., 2013). These differing results may be due to differences in age, country, and measures between studies. First, most studies showing an increase in the prevalence of depression or depressive symptoms were done among participants in their late midlife (Jeuring et al., 2018) or at least included also younger participants (Bishop et al., 2022; Spiers et al., 2012). Based on a national FinHealth study, the prevalence of depression has increased also in Finland between 2011 and 2017, but mainly among working-age adults (Suvisaari et al., 2018). Second, the earlier-born cohorts in the present study (born in 1910 and 1914) participated in World War II as young adults and lived in the post-war reconstruction. This may explain the differences to otherwise relatively similar neighboring country Sweden, for example, in which the prevalence of minor depression increased among 75-year-old cohorts at the beginning of the 2000s compared to 30 years earlier (Wiberg et al., 2013). Third, CES-D was designed to assess depressive symptomology in the general population, not for clinical diagnostic purposes (Radloff, 1977). Even though the items overlap with DSM-IV diagnostic criteria for depression, it does not assess the prevalence of depression. Forth, understanding, diagnosis, and treatment of depression have increased over the last decades (Herrman et al., 2019), and the decrease in depressive symptoms in the present study may be partly a result of better prevention and treatment of depressive symptoms in the later-born cohorts.

Interestingly, the cohort differences in life satisfaction were seen only with the item evaluating life satisfaction until now and not with the current life satisfaction. This is in line with the “satisfaction paradox” suggesting a relatively high level of satisfaction despite relatively bad conditions (Walker, 2005) and previous studies showing no cohort differences in overall life satisfaction (Henning et al., 2022; Jivraj et al., 2014). The standards of good health may change over time (Jylhä, 2009), and similarly, the standards for life satisfaction may change over time. Life satisfaction can be seen as a result of a match between subjective standards for a good life and reality (Schilling, 2005; Veenhoven, 1996) and the older generations may have lower expectations in relation to their age than younger ones (Walker, 2005). The standards may be a result of comparing life circumstances to others living in the same circumstances (Diener & Fujita, 1997) and thus, the improved life circumstances do not affect life satisfaction as also the reference group has the same circumstances (Henning et al., 2022). Individuals may also compare their current situation to their past life (Diener & Fujita, 1997; Schilling, 2005), which may explain why 80-year-old men in the earlier-born cohort were even more likely to be satisfied with their current situation than the later-born cohort. The earlier-born cohort of 80-year-old men had survived many hardships during their life, such as wars, which may underlie satisfaction with the current more secure living conditions that they experienced in 1989–1990. However, 80-year-old men are also the ones whose survival rate increased the most during 28 years: 31% of men in the earlier-born cohort and 57% of men in the later-born cohort were alive at age 80 (Statistics Finland, 2022). The earlier-born men may have been satisfied with their current situation because they were still alive unlike most of their age cohort. Men in the later-born cohort had a different situation, as they may have survived with more diseases and health limitations and a larger proportion of their age peers were alive at age 80.

There might be various explanations behind the found cohort differences. As mentioned above, the earlier-born cohort lived through the independence of Finland (1917), the Civil War (1918), the Winter War (1939–40), the Continuation War (1941–1944), and the Lapland War (1944–1945), and thus lived through an epoch with several episodes of hardship beyond control. The later-born cohorts were born during wartime, but they grow up during the period of rapid development and the occurrence of many social reforms in Finland. In all, their circumstances have been more foreseeable than the earlier cohort’s. However, it is not possible to separate the effects of birth cohort and period from each other and it is likely that many societal changes have produced both period effects affecting all age groups simultaneously as well as cohort effects affecting specific age cohorts (Bell, 2020).

Better self-rated health in the later-born cohorts partly explained the cohort differences in depressive symptoms (especially in somatic symptoms), and in satisfaction with the past life. In addition, higher education in the later-born cohorts partly explained the cohort differences. However, most of the cohort differences were not completely explained by the covariates included in the present study. Previous studies have shown that later-born cohorts report more internal control beliefs, less external control beliefs (Gerstorf et al., 2019; Hülür et al., 2016), and perceive fewer constraints in their life (Drewelies et al., 2018) than earlier cohorts. These findings suggest that later-born cohorts perceive themselves to be more in charge of what happens in their life, which in turn may lead to a lower risk of depressive symptoms (Presson & Benassi, 1996). Altogether, it is likely that various societal changes have led to changes in an individual´s physical, cognitive, and psychological functioning, which in turn lead to cohort and period differences in mental well-being. There might also be period effects that are specific to the Finnish population as experiencing positive feelings has increased during the last 15 years in Finland and reached the level of other Nordic countries while experiencing negative feelings has been quite stable in Finland but increased in other European countries (Helliwell et al., 2022).

A major strength of this study is the use of identical methods 28 years apart. Participant recruitment, mental well-being questionnaires, and questions and performance assessments were comparable between cohorts. This is one of the first studies focusing on mental well-being in relatively old age and including both negative (depressive symptoms) and positive (life satisfaction) aspects of mental well-being in the same study. In addition, this study contributes to the literature by investigating not only the cohort differences but also the underlying factors behind these differences.

Some limitations should be kept in mind when considering the findings of this study. The participation rate was lower in the later than in the earlier cohort study, which may suggest that the later cohort may be more selected, and the results can be partly explained by selection bias. However, it should be taken into account that the earlier cohort was already more selected. Around 60% of the Finnish people born in 1914 were alive at age 75, while the corresponding proportion for people born in 1942–1943 was 76% (Statistics Finland, 2022). The corresponding proportions at age 80 were 45% for people born in 1910 and 65% for people born in 1938–1939. In the later-born cohort also, individuals with diseases and health limitations are more likely to survive to old age which may attenuate the cohort differences. The reasons for declining to participate in the study did not differ materially between cohorts (Koivunen et al., 2021), and also the prevalence of high depressive symptoms (CES-D score ≥20, (Vilagut et al., 2016)) in the later cohort study is rather similar to the prevalence of high depressive symptoms in Finnish older adults in general (Suvisaari et al., 2018). Thus, the cohorts may be assumed to be comparable. The information about antidepressant use was not comparable across cohorts and was not controlled for in the analysis. While the CES-D is a validated questionnaire, the assessment of life satisfaction was not optimal. Both current life satisfaction and life satisfaction until now were assessed with single items and rather rough response scales. With self-reported assessments, it is also not possible to exclude the possible changes in the understanding or perception of the constructs over time and between cohorts. However, life satisfaction and depressive symptoms are subjective experiences, and individuals themselves are the best evaluators of their feelings and experiences.

To conclude, this study provides evidence for improved mental well-being in the more recent birth cohorts of 75- and 80-year-old Finnish adults. Better self-rated health and higher education of the later-born cohort explained the cohort differences only partly. While satisfaction with the past life and the lack of depressive symptoms are important outcomes as such, they are also important resources to function in society and daily life. Moreover, both positive feelings and the lack of depressive symptoms predict longevity, better health, and better functioning (Diener & Chan, 2011; Formánek et al., 2020; Kokko & Feldt, 2018). Future research should address whether this positive change continues among the next cohorts.

## Supplemental Material

Supplemental Material - Cohort Differences in Depressive Symptoms and Life Satisfaction in 75- and 80-Year-Olds: A Comparison of Two Cohorts 28 Years Apart Click here for additional data file. Supplemental Material for Cohort Differences in Depressive Symptoms and Life Satisfaction in 75- and 80-Year-Olds: A Comparison of Two Cohorts 28 Years Apart by Tiia Kekäläinen, Kaisa Koivunen, Katja Pynnönen, Erja Portegijs, and Taina Rantanen in Journal of Aging and Health.
