# Supplementary material for: Cohort Differences in Depressive Symptoms and Life Satisfaction in 75- and 80-Year-Olds: A Comparison of Two Cohorts 28 Years Apart
Source: J Aging Health. 2023 Mar 22;36(1-2):3–13. doi: 10.1177/08982643231164739 (PMC10693740; doi:10.1177/08982643231164739)
Supplement: Supplemental Material - Cohort Differences in Depressive Symptoms and Life Satisfaction in 75- and 80-Year-Olds: A Comparison of Two Cohorts 28 Years Apart [file sj-pdf-1-jah-10.1177_08982643231164739.pdf]

Kekäläinen, T., Koivunen, K., Pynnönen, K., Portegijs, E., & Rantanen, T.
Cohort differences in depressive symptoms and life satisfaction in 75- and 80-year-olds: a comparison of two cohorts 28 years apart

Supplementary Materials

Table S1. Regression coefficients of the association between the birth cohort and the Center for Epidemiological Studies Depression Scale dimensions in men

	Men 75 years				Men 80 years			
	Birth cohort			Model	Birth cohort			Model
	B	SE	p	Adj R^2	B	SE	p	Adj R^2
**Depressed affect**								
Birth cohort	-1.59	.32	<.001	.07	-1.96	.53	<.001	.06
+Education	-1.59	.40	<.001	.07	-1.50	.63	.018	.06
+Marriage	-1.57	.32	<.001	.11	-1.74	.53	.001	.08
+Social contacts	-1.57	.34	<.001	.07	-1.87	.60	.002	.06
+Loneliness	-1.10	.29	<.001	.27	-2.31	.48	<.001	.25
+Self-rated health	-1.13	.35	.001	.11	-.95	.54	.078	.16
+All	-.90	.39	.021	.27	-1.24	.67	.066	.29
**Lack of positive affect**								
Birth cohort	-2.66	.35	<.001	.16	-2.51	.53	<.001	.10
+Education	-1.93	.43	<.001	.18	-1.82	.62	.004	.11
+Marriage	-2.64	.35	<.001	.18	-2.26	.53	<.001	.12
+Social contacts	-2.64	.37	<.001	.15	-2.31	.58	<.001	.12
+Loneliness	-2.40	.35	<.001	.19	-2.87	.51	<.001	.19
+Self-rated health	-1.99	.38	<.001	.20	-1.91	.53	<.001	.19
+All	-1.33	.45	.004	.24	-1.52	.68	.026	.28
**Interpersonal problems**								
Birth cohort	-.07	.10	.498	.00	-.65	.17	<.001	.06
+Education	.00	.12	.992	.00	-.52	.20	.012	.06
+Marriage	-.06	.10	.510	.00	-.62	.18	<.001	.06
+Social contacts	-.06	.10	.553	.00	-.63	.19	.001	.06
+Loneliness	-.05	.10	.626	.00	-.68	.18	<.001	.06
+Self-rated health	.00	.11	.985	.00	-.60	.18	.001	.08
+All	.04	.13	.741	.00	-.49	.25	.049	.06
**Somatic symptoms**								
Birth cohort	-1.28	.37	.001	.03	-1.13	.63	.076	.01
+Education	-1.32	.46	.004	.03	-.99	.75	.185	.01
+Marriage	-1.27	.37	.001	.04	-.86	.63	.174	.03
+Social contacts	-1.20	.39	.002	.04	-.95	.70	.181	.02
+Loneliness	-.92	.36	.012	.11	-1.58	.58	.007	.19
+Self-rated health	-.50	.40	.213	.10	.23	.62	.713	.16
+All	-.44	.47	.359	.15	-.35	.78	.658	.27

Note. B = Unstandardized beta indicates mean cohort difference (reference group Evergreen cohort), SE = standard error, Adj R^2 = adjusted R^2. Each covariate was added in the model one at a time with birth cohort and all six together in the model "All".

Table S2. Regression coefficients of the association between the birth cohort and the Center for Epidemiological Studies Depression Scale dimensions in women

	Women 75 years				Women 80 years			
	Birth cohort			Model	Birth cohort			Model
	B	SE	p	Adj R^2	B	SE	p	Adj R^2
**Depressed affect**								
Birth cohort	-1.95	.32	<.001	.07	-1.82	.38	<.001	.06
+Education	-1.60	.42	<.001	.07	-2.14	.45	<.001	.06
+Marriage	-1.89	.34	<.001	.07	-1.63	.38	<.001	.07
+Social contacts	-1.95	.34	<.001	.08	-1.64	.43	<.001	.07
+Loneliness	-1.90	.30	<.001	.24	-2.06	.37	<.001	.17
+Self-rated health	-1.13	.35	.001	.15	-.99	.38	.010	.15
+All	-1.21	.42	.004	.29	-1.42	.53	.008	.23
**Lack of positive affect**								
Birth cohort	-2.91	.26	<.001	.20	-2.86	.37	<.001	.15
+Education	-2.80	.34	<.001	.20	-3.15	.44	<.001	.15
+Marriage	-2.88	.28	<.001	.20	-2.65	.38	<.001	.16
+Social contacts	-2.91	.28	<.001	.21	-2.67	.42	<.001	.16
+Loneliness	-2.92	.26	<.001	.24	-2.97	.37	<.001	.18
+Self-rated health	-2.26	.28	<.001	.25	-2.13	.38	<.001	.22
+All	-2.40	.37	<.001	.27	-2.14	.54	<.001	.23
**Interpersonal problems**								
Birth cohort	-.20	.09	.020	.01	-.42	.09	<.001	.06
+Education	-.10	.11	.365	.01	-.41	.11	<.001	.05
+Marriage	-.18	.09	.045	.01	-.42	.10	<.001	.05
+Social contacts	-.20	.09	.028	.01	-.40	.11	<.001	.06
+Loneliness	-.18	.09	.033	.04	-.44	.09	<.001	.07
+Self-rated health	-.11	.10	.246	.02	-.34	.10	.001	.07
+All	-.04	.12	.749	.04	-.36	.14	.011	.06
**Somatic symptoms**								
Birth cohort	-1.03	.31	.001	.02	-1.31	.39	.001	.03
+Education	-1.06	.40	.008	.02	-1.50	.47	.001	.03
+Marriage	-1.10	.33	.001	.02	-1.20	.41	.004	.03
+Social contacts	-1.04	.32	.002	.03	-1.12	.45	.013	.04
+Loneliness	-1.04	.31	.001	.07	-1.43	.40	<.001	.06
+Self-rated health	-.19	.33	.566	.11	-.49	.40	.224	.11
+All	-.58	.42	.173	.14	-.56	.57	.329	.12

Note. B = Unstandardized beta indicates mean cohort difference (reference group Evergreen cohort), SE = standard error, Adj R^2 = adjusted R^2. Each covariate was added in the model one at a time with birth cohort and all five together in the model “All”.

Table S3. Regression coefficients of the association between birth cohort and mental well-being outcomes in men who participated in the laboratory measures (n = 479)

	Men 75 years				Men 80 years			
	Birth cohort			Model	Birth cohort			Model
	B	SE	p	Adj R2	B	SE	p	Adj R2
Depressive symptoms								
Birth cohort	-4.89	.79	<.001	.12	-4.96	1.24	<.001	.08
+Walking speed	-4.21	.79	<.001	.16	-3.83	1.27	.003	.11
+Cognitive functioning	-4.11	.86	<.001	.16	-2.64	1.35	.051	.14
+All	-2.62	1.10	.019	.28	-3.25	1.85	.081	.35
Current life satisfaction								
Birth cohort	-.02	.45	.971	.00	-1.38	.67	.049	.04
+Walking speed	-.29*	.47	.539	.06	-1.98	.72	.007	.16
Birth cohort	.25	.42	.553	.00	-1.22	.65	.059	.04.
+Cognitive functioning	-.09	.47	.842	.02	-1.81	.72	.013	.09
Birth cohort	-.04	.48	.931	.00	-1.45	.79	.065	.04
+All	-.48	.61	.437	.20	-2.04	1.00	.040	.38
Life satisfaction until now								
Birth cohort	1.55	.36	<.001	.12	1.65	.42	<.001	.14
+Walking speed	1.49	.37	<.001	.12	1.55	.43	<.001	.15
Birth cohort	1.63	.36	<.001	.13	1.74	.43	<.001	.15
+Cognitive functioning	1.40	.40	<.001	.14	1.41	.48	.003	.17
Birth cohort	1.68	.38	<.001	.12	2.14	.52	<.001	.18
+All	.51**	.50	.312	.19	1.73	.72	.016	.31

Note. Depressive symptoms analyzed with linear regression and life satisfaction with binary logistic regression. B = Unstandardized beta / KHB logistic regression coefficient indicates mean cohort difference (reference group Evergreen cohort), SE = standard error, Adj R^2 = adjusted R^2 (linear regression) or Pseudo R^2 (binary regression). *p<.05, **p<.01, ***p<.001 indicating a statistically significant difference in KHB logistic regression coefficients between a model with only a birth cohort and a model with covariate(s). Each covariate was added in the model one at a time with birth cohort. The model “All” includes walking speed, cognitive functioning, education, marital status, social contacts, loneliness, and self-rated health.

Table S4. Regression coefficients of the association between birth cohort and mental well-being outcomes in women who participated in the laboratory measures (n = 746)

	Women 75 years				Women 80 years			
	Birth cohort			Model	Birth cohort			Model
	B	SE	p	Adj R2	B	SE	p	Adj R2
Depressive symptoms								
Birth cohort	-5.77	.76	<.001	.12	-5.94	.94	<.001	.12
+Walking speed	-5.08	.81	<.001	.13	-4.03	1.04	<.001	.17
+Cognitive functioning	-3.91	.94	<.001	.14	-3.12	1.17	.008	.17
+All	-3.91	1.02	.001	.30	-3.37	1.43	.019	.29
Current life satisfaction								
Birth cohort	.28	.35	.987	.00	.08	.38	.839	.00
+Walking speed	-.01*	.37	.033	03	-.71***	.44	.107	.10
Birth cohort	.30	.35	.394	.00	.36	.36	.307	.01
+Cognitive functioning	-.32*	.44	.465	.03	-.62**	.45	.172	.09
Birth cohort	.17	.40	.676	.00	-.03	.44	.938	.00
+All	-.90*	.59	.127	.26	-1.45**	.64	.023	.26
Life satisfaction until now								
Birth cohort	1.18	.27	<.001	.08	1.60	.32	<.001	.14
+Walking speed	1.03	.28	<.001	.09	1.32	.36	<.001	.15
Birth cohort	1.19	.27	<.001	.08	1.45	.33	<.001	.12
+Cognitive functioning	.85	.33	.009	.09	1.21	.39	.002	.12
Birth cohort	1.33	.29	<.001	.09	1.53	.37	<.001	.11
+All	.46**	.40	.245	.19	1.41	.52	.006	.19

Note. Depressive symptoms analyzed with linear regression and life satisfaction with binary logistic regression. B = Unstandardized beta / KHB logistic regression coefficient indicates mean cohort difference (reference group Evergreen cohort), SE = standard error, Adj R^2 = adjusted R^2 (linear regression) or Pseudo R^2 (binary regression). *p<.05, **p<.01, ***p<.001 indicating a statistically significant difference in KHB logistic regression coefficients between a model with only a birth cohort and a model with covariate(s). Each covariate was added in the model one at a time with birth cohort. The model "All" includes walking speed, cognitive functioning, education, marital status, social contacts, loneliness, and self-rated health.

Table S5. Regression coefficients of the association between the birth cohort and the Center for Epidemiological Studies Depression Scale dimensions in men who participated in the laboratory measures (n = 479)

	Men 75 years				Men 80 years			
	Birth cohort			Model	Birth cohort			Model
	B	SE	p	Adj R^2	B	SE	p	Adj R^2
**Depressed affect**								
Birth cohort	-1.30	.28	<.001	.07	-1.55	.45	.001	.06
+Walking speed	-1.08	.28	<.001	.11	-1.21	.47	.010	.08
+Cognitive functioning	-1.06	.31	.001	.08	-1.02	.49	.040	.08
+All	-.79	.33	.017	.25	-.67	.55	.230	.28
**Lack of positive affect**								
Birth cohort	-2.45	.32	<.001	.17	-2.05	.45	<.001	.10
+Walking speed	-2.28	.32	<.001	.19	-1.65	.46	.001	.13
+Cognitive functioning	-2.07	.35	<.001	.19	-1.48	.49	.003	.13
+All	-1.30	.41	.002	.24	-1.20	.57	.036	.28
**Interpersonal problems**								
Birth cohort	-.05	.09	.589	.00	-.43	.15	.004	.04
+Walking speed	.01	.09	.924	.02	-.33	.15	.034	.06
+Cognitive functioning	.02	.10	.846	.00	-.25	.16	.127	.07
+All	.04	.12	.717	.01	-.19	.20	.349	.06
**Somatic symptoms**								
Birth cohort	-1.10	.33	.001	.04	-.95	.51	.064	.01
+Walking speed	-.90	.34	.008	.06	-.63	.53	.235	.03
+Cognitive functioning	-1.08	.37	.004	.03	-.31	.55	.577	.05
+All	-.52	.42	.216	.12	-.10	.63	.877	.24

Note. B = Unstandardized beta indicates mean cohort difference (reference group Evergreen cohort), SE = standard error, Adj R^2 = adjusted R^2. Each covariate was added in the model one at a time with birth cohort. The model “All” includes walking speed, cognitive functioning, education, marital status, loneliness, and self-rated health.

Table S6. Regression coefficients of the association between the birth cohort and the Center for Epidemiological Studies Depression Scale dimensions in women who participated in the laboratory measures (n = 746)

	Women 75 years				Women 80 years			
	Birth cohort			Model	Birth cohort			Model
	B	SE	p	Adj R^2	B	SE	p	Adj R^2
**Depressed affect**								
Birth cohort	-1.86	.32	<.001	.07	-1.67	.39	<.001	.06
+Walking speed	-1.61	.35	<.001	.08	-.96	.42	.022	.10
+Cognitive functioning	-1.14	.40	.004	.09	-1.03	.45	.023	.08
+All	-.83	.41	.042	.27	-.70	.52	.179	.21
**Lack of positive affect**								
Birth cohort	-2.74	.26	<.001	.21	-2.57	.36	<.001	.15
+Walking speed	-2.64	.28	<.001	.21	-2.04	.41	<.001	.17
+Cognitive functioning	-2.35	.32	<.001	.21	-2.27	.44	<.001	.15
+All	-2.02	.35	<.001	.28	-1.66	.52	.001	.24
**Interpersonal problems**								
Birth cohort	-.17	.08	.046	.01	-.41	.09	<.001	.06
+Walking speed	-.14	.09	.114	.01	-.37	.12	<.001	.06
+Cognitive functioning	-.06	.10	.589	.01	-.29	.11	.009	.07
+All	-.02	.12	.874	.05	-.27	.14	.049	.07
**Somatic symptoms**								
Birth cohort	-1.03	.31	.001	.02	-1.29	.38	.001	.04
+Walking speed	-.69	.33	.036	.04	-.64	.49	.134	.07
+Cognitive functioning	-.64	.38	.094	.03	-.83	.46	.073	.04
+All	-.46	.42	.268	.13	-.13	.56	.817	.11

Note. B = Unstandardized beta indicates mean cohort difference (reference group Evergreen cohort), SE = standard error, Adj R^2 = adjusted R^2. Each covariate was added in the model one at a time with birth cohort. The model "All" includes walking speed, cognitive functioning, education, marital status, loneliness, and self-rated health.
